# Risk-Based Screening for Thyroid Dysfunction during Pregnancy

**DOI:** 10.1155/2013/619718

**Published:** 2013-03-27

**Authors:** Masanao Ohashi, Seishi Furukawa, Kaori Michikata, Katsuhide Kai, Hiroshi Sameshima, Tsuyomu Ikenoue

**Affiliations:** ^1^Department of Obstetrics & Gynecology, Miyazaki Medical Association Hospital, 738-1 Shinbepputyo-Funato, Miyazaki 880-0834, Japan; ^2^Department of Obstetrics & Gynecology, Faculty of Medicine, University of Miyazaki, 5200 Kihara-Kiyotake, Miyazaki 889-1692, Japan

## Abstract

*Objective*. We conducted the study to see the incidence of thyroid dysfunction in women with obstetrical high-risk factors. *Methods*. We retrospectively reviewed medical charts of high-risk pregnant women who had examination for thyroid function during pregnancy. Women were divided according to clinical presentation, symptoms of thyroid disease and those with a personal history of thyroid disease (thyroid disease, *n* = 32), intrauterine growth restriction (IUGR, *n* = 115), diabetes mellitus (diabetes, *n* = 115), hypertension (*n* = 63), intrauterine fetal death (IUFD, *n* = 52), and placental abruption (abruption, *n* = 15). The incidence of thyroid dysfunctions including hyperthyroidism or hypothyroidism was compared. *Results*. The overall prevalence of thyroid dysfunction was 24.7%. The incidence of thyroid dysfunction in each group was as follows: 31% in thyroid disease, 25% in IUGR, 30% in diabetes, 27% in hypertension, 12% in IUFD, and 7% in abruption. Except IUFD, the incidence was not statistically significant from the group of thyroid disease (thyroid disease versus IUFD, *P* = 0.03 by *χ* ^2^ test). Thyroid disease represented for only 10% of all thyroid dysfunctions. *Conclusion*. Testing of women with a personal history or current symptoms of thyroid disease during pregnancy may be insufficient to detect women with thyroid dysfunction, who will become at high-risk pregnancy.

## 1. Introduction

Thyroid dysfunction is present in 2.3–3.8% in general [1], as well as during pregnancy [2]. However, nearly 10% of abnormal thyroid function was observed according to thyroid function survey using general health checkup system for the adult in Japan [3]. This implicates that pregnant women in Japan also have higher prevalence of thyroid dysfunction. Up to now, there are no large population studies regarding the prevalence of thyroid dysfunction in pregnant women in Japan.

Thyroid dysfunctions are associated with neuropsychological complications on child. A well-known complication is low intelligence quotient in the infants born from mothers with overt hypothyroidism [4]. Subclinical hypothyroidism is also associated with impaired neuropsychological development of children [5]. Indeed, it remains unclear whether to treat subclinical hypothyroidism or not during pregnancy [6]. Thyroid dysfunctions are also associated with several obstetrical complications such as preeclampsia and placental abruption [7–10]. Consequently, there is a question whether the management of thyroid dysfunction could reduce these complications on both mother and child during pregnancy.

Currently, no consensus has been reached on the universal screening for thyroid function for pregnant women. ACOG recommended thyroid function screening during pregnancy should be limited to women with symptoms of thyroid disease and those with a history of thyroid disease or other medical conditions associated with it [11]. However, this conventional screening may be insufficient because most of pregnant women that have thyroid dysfunction are asymptomatic and without a history of thyroid disease. Therefore, consideration should be given to extend the screening objects including women with possible thyroid disease except for women with symptoms of thyroid disease and those with a history of thyroid disease.

Here, we conducted a study to see the incidence of thyroid dysfunction in pregnant women having obstetrical or medical complications. If we obtain a high incidence of thyroid dysfunction in some high-risk groups, a more reasonable approach for screening would be provided.

## 2. Materials and Methods

This study was undertaken retrospectively and there was no need to be approved by a suitably constituted Ethics Committee of our institution. We retrospectively reviewed the medical charts of women with obstetrical or medical complications that were admitted to the Perinatal Center of Miyazaki Medical Association Hospital from January 2001 to April 2011. Our hospital mainly deals with referral cases in the central part of Miyazaki province, Japan. The total number of deliveries was 4381 in the study period. In Miyazaki province, when antepartum high-risk factors are diagnosed, women are advised to visit high-level perinatal centers where they finally deliver their babies. High-risk factors include prenatal medical complications such as diabetes, obstetric complications such as hypertensive disorders, and fetal complications such as growth restriction. Additionally, some emergencies may occur and they are transferred to the 8 high-level centers. As a consequence, 80% of total deliveries (pregnant women without risk) were carried out in private clinics. The rest deliveries with high-risk factors were carried out in 8 high-level centers [12]. Our perinatal center is one of them.

Pregnant women with the following complications were selected: women with symptoms of thyroid disease and those with a personal history of thyroid disease (thyroid disease), intrauterine growth restriction (IUGR), diabetes mellitus (diabetes), hypertension, intrauterine fetal death (IUFD), and placental abruption (abruption). The signs and symptoms of hyperthyroidism included tremors, nervousness, insomnia, excessive sweating, heat intolerance, tachycardia, hypertension, and goiter. The signs and symptoms of hypothyroidism included fatigue, muscle cramps, constipation, cold intolerance, and hair loss. The history of thyroid disease was either known hyperthyroidism such as Graves' disease or hypothyroidism such as Hashimoto thyroiditis, subacute thyroiditis, and iodine deficiency. IUGR was defined as sex-specific birth weight less than the 10th percentile for gestational age according to the Japanese standard growth curve for singletons [13]. Diabetes was defined as either preexisting diabetes mellitus or gestational diabetes mellitus based on 75 g glucose tolerance test. Gestational diabetes mellitus is diagnosed, if one or more of these readings are elevated in this study; at fasting ≥100 mg/dL, at 1-hour ≥180 mg/dL and at 2-hour ≥150 mg/dL [14]. Hypertension included preeclampsia, gestational hypertension, and chronic hypertension. Preeclampsia was diagnosed when hypertension (systolic ≥140 mmHg or ≥90 mmHg diastolic) and proteinuria (dipstick ≥2+) developed after 20 weeks of gestation. Gestational hypertension was diagnosed when hypertension (systolic ≥140 mmHg or ≥90 mmHg diastolic) without proteinuria developed after 20 weeks of gestation. Chronic hypertension was defined as hypertension diagnosed prior to conception or within the first 20 weeks of pregnancy. Placental abruption was determined by the presence of retroplacental hematoma and clinical presentations (any one or combination of genital bleeding, abdominal pain, pregnancy-induced hypertension, premature labor, premature rupture of membrane, IUFD, or nonreassuring fetal status) [15]. In case of placental abruption with pregnancy-induced hypertension or IUFD, the case was categorized as placental abruption. A total of 838 pregnancies displaying obstetrical or medical complications were found in the study period.

From the 838 pregnancies, we selected the 392 cases that had examined thyroid function test from 1st to 3rd trimesters. They were IUGR (*n* = 115), diabetes (*n* = 115), hypertension (*n* = 63), IUFD (*n* = 52), abruption (*n* = 15), and thyroid disease (*n* = 32).

**Figure 1:**
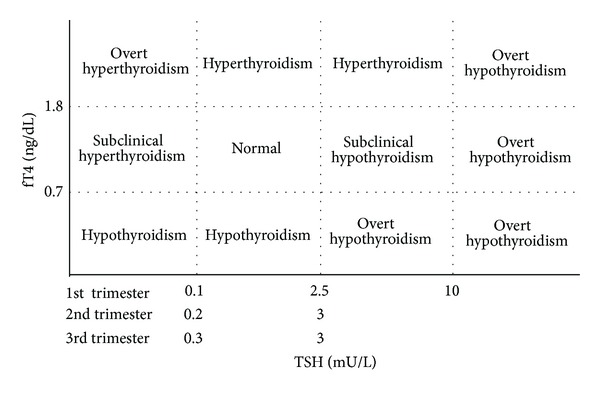
The definition of thyroid dysfunction in this study.

The test of thyroid function was done in our hospital. Serum concentrations of TSH and free T4 (fT4) were measured by electro chemiluminescence immunoassay or chemiluminescent enzyme immunoassay (Abbott Japan, ARCHITECT TSH, fT4). The definition of thyroid dysfunction was followed (Figure 1). Hyperthyroidism was defined as low TSH (<0.10 mU/L in 1st trimester, <0.20 mU/L in 2nd, and <0.30 mU/L in 3rd trimester) and normal-to-high fT4 (>0.7 ng/dL). Hypothyroidism was defined as high TSH (>2.5 mU/L in 1st trimester, >3.0 in 2nd and 3rd trimesters) and normal-to-low fT4 (<1.8 ng/dL). Low fT4 (<0.7 ng/dL) with normal-to-low TSH was categorized as hypothyroidism. High fT4 (>1.8 ng/dL) with normal-to-high TSH was categorized as hyperthyroidism. Furthermore, we divided thyroid dysfunctions into the following subgroups. Overt hypothyroidism was defined as high TSH (≧2.5 mU/L) in conjunction with low fT4 (<0.7 ng/dL) or high TSH (≧10.0 mU/L) irrespective of fT4 levels. Overt hyperthyroidism was defined as low TSH (<0.1 mU/L) in conjunction with high fT4 (≧1.8 ng/dL). Subclinical hypothyroidism was defined as high TSH (2.5–10.0 mU/L) in conjunction with normal fT4. Subclinical hyperthyroidism was defined as low TSH (<0.1 mU/L) in conjunction with normal fT4. Thyroid dysfunctions without fulfilling the above criteria of subgroups were categorized as other. The alterations in thyroid function during pregnancy can pose challenges to the interpretation of laboratory thyroid tests. Therefore, we used trimester-specific reference intervals for thyroid function, following the guidelines of the American Thyroid Association [16].

The following clinical characteristics were also collected: maternal age, parity, gestational age at examination of thyroid function (weeks), gestational age at delivery (weeks), birth weight (g), and cesarean delivery. Perinatal outcomes were investigated and included evaluations of umbilical artery pH (UA pH) and neonatal death (ND). The incidence of thyroid dysfunctions including hyperthyroidism or hypothyroidism was examined and compared among the groups of IUGR, diabetes, hypertension, IUFD, abruption, and thyroid disease.

Comparisons were made using *χ* ^2^ tests. Data are expressed as number, incidence (%), or mean ± SD. Probability values <0.05 were considered significant.

## 3. Results

In the 392 women, the mean gestational age at delivery was 34.3 ± 6.3 weeks (Table 1). Nulliparous pregnancies constituted 48.5%, and the cesarean delivery rate was 44.9%. There were no neonatal deaths. In 73.2% of cases, thyroid function was examined at third trimester.

**Figure 2:**
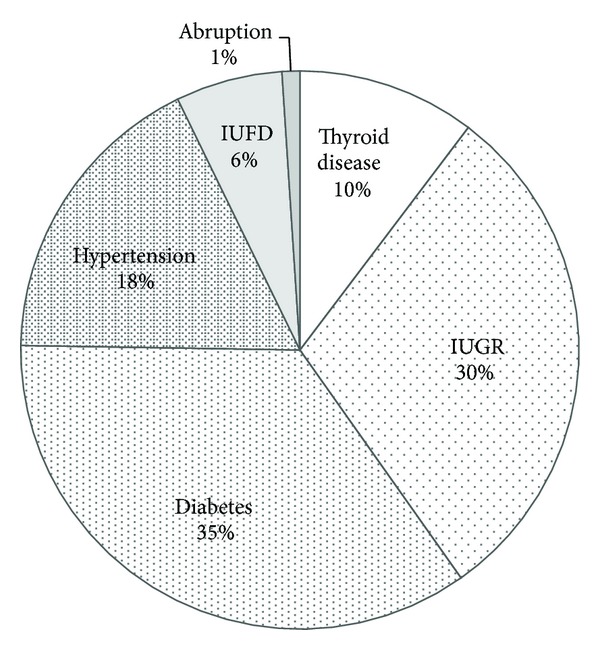
The prevalence of each group in thyroid dysfunction. Thyroid disease: women with symptoms of thyroid disease and those with a history of thyroid disease, IUGR: intrauterine growth restriction, IUFD: intrauterine fetal death, and abruption: placental abruption.

We found 97 women of thyroid dysfunctions in the 392 study subjects. The incidence of thyroid dysfunction was 24.7%. The incidence of thyroid dysfunction of each group is as follows: 31% in thyroid disease, 25% in IUGR, 30% in diabetes, 27% in hypertension, 12% in IUFD, and 7% in abruption (Table 2). The incidence of thyroid dysfunction was statistically insignificant in IUGR, diabetes, HT, and abruption. The group of thyroid disease accounted for only 10% of all thyroid dysfunctions (Figure 2).

Hypothyroidism was more prevalent than hyperthyroidism in the study groups except for the thyroid disease group. Overt hyperthyroidism was not found in any study group. On the other hand, three cases of overt hypothyroidism were found in the groups of diabetes and HT. 26 cases of subclinical hyperthyroidism and 55 cases of subclinical hypothyroidism were found in the study groups (Table 2).

## 4. Discussion

The incidences of thyroid dysfunction in the diabetes (30%) and thyroid disease (31%) were slightly higher than those of other groups as previously reported [17, 18]. On the other hand, the incidences of thyroid dysfunction in IUGR (25%) and hypertension (27%) were also high. Besides, this study showed that the screening for women with thyroid disease (symptoms of thyroid disease and personal history of thyroid disease) could pick up only 10% of affected women. In other words, it would miss 90% of affected women with thyroid dysfunction (Figure 1). Therefore, current screening strategy for thyroid dysfunction during pregnancy is not sufficient.

In our study, hypothyroidism was more prevalent than hyperthyroidism and most of cases ware categorized as subclinical disease (Table 2). In contrast to overt diseases, subclinical hypothyroidism and subclinical hyperthyroidism are not associated with poor pregnancy outcomes [19, 20]. However, apart from preterm delivery or miscarriage, it was also reported that major obstetrical complications such as hypertension, IUFD, or abruption were closely associated with subclinical hypothyroidism [21–23]. Recently, a close association between gestational diabetes mellitus and subclinical hypothyroidism was reported [24]. We also found that the incidence of thyroid dysfunction in the DM group was similar to the groups of thyroid disease, IUGR, and hypertension. The DM group in our study mainly consisted of gestational diabetes mellitus. High frequency of antithyroid antibodies in pregnant women with gestational diabetes mellitus was also reported [18]. ACOG and The Endocrine Society recommended that thyroid testing should be limited to women with symptoms of thyroid disease and those with a history of thyroid disease or other medical conditions associated with it, such as type 1 diabetes or autoimmune disorders [11, 25]. According to our results, we recommended to screen thyroid function in women having the above-mentioned complications. Furthermore, three cases of overt hypothyroidism were noticed in the groups of DM and HT. We were not able to detect three cases of overt hypothyroidism, if thyroid testing was limited to women with thyroid disease.

We found high incidence of thyroid dysfunction in our study. One of the reasons was the trimester-specific range for TSH in this study. The normal range of TSH in general population is 0.45 to 4.5 mU/L. The normal range of TSH during pregnancy is narrow when compared to that of general population. Another reason was our study population. Our perinatal center is a secondary hospital and mainly deals with referral cases due to thyroid disease, IUGR, diabetes mellitus, hypertension, IUFD, and placental abruption. These complications were frequently associated with hypothyroidism rather than hyperthyroidism [21–23]. The timing of thyroid screening should also be taken into account. High hCG level at first trimester resulted in hCG-induced hyperthyroidism. In our study, first trimester screening constituted 5.0% only (Table 1).

This study had some limitations. First, the current study was done in one secondary hospital dealing with referral cases only. As a consequence, there was a higher proportion of high-risk deliveries and a higher incidence of cesarean deliveries in our hospital. A regional population-based study should be needed to verify the prevalence of thyroid dysfunction and the relevance of perinatal prognosis in low-risk population. Second, we did not investigate thyroid function of the child who was born from a mother with thyroid dysfunction. The outcome of infant should be obtained to verify the effect of thyroid dysfunction during pregnancy. Third, we partially measured antithyroid antibodies for thyroid dysfunction cases. The presence of antibodies influences clinical course of pregnancy independently from thyroid dysfunction [25].

In conclusion, we demonstrated that the prevalence of thyroid dysfunction is increased in pregnant women with obstetrical or medical complications. Under the currently recommended screening method, the majority of thyroid dysfunctions may be missing. With a full awareness of high incidence of thyroid dysfunction in pregnant women with obstetrical or medical complications, consideration should be given regarding the screening efficiency during pregnancy.

## Figures and Tables

**Table 1:** Demographic data of study groups.

Maternal age (years)	30.8 ± 4.7
Nulliparity	190 (48.5%)
Gestational age at examination	
1st trimester	20 (5.1%)
2nd trimester	85 (21.7%)
3rd trimester	287 (73.2%)
Gestational age at delivery	34.3 ± 6.3
Cesarean delivery	176 (44.9%)
pH of umbilical artery	7.28 ± 0.55
Neonatal death	0

**Table 2:** Incidence of thyroid dysfunction in study groups.

Study groups (*n*)	Thyroid disease 32	IUGR 115	Diabetes 115	Hypertension 63	IUFD 52	Abruption 15	Total 392
Thyroid dysfunction							
(*n*)	10	29	34	17	6^a^	1	97
(%)	(31)	(25)	(30)	(27)	(12)	(7)	
Hypothyroidism							
Overt	0	0	2	1	0	0	3
Subclinical	5	19	15	11	4	1	55
Other	0	2	4	2	0	0	8
Hyperthyroidism							
Overt	0	0	0	0	0	0	0
Subclinical	5	5	12	3	1	0	26
Other	0	3	1	0	1	0	5

^a^ *P* = 0.03 versus thyroid disease by *χ* ^2  ^ test.

## References

[1] Leese GP, Flynn RV, Jung RT, MacDonald TM, Murphy MJ, Morris AD (2008). Increasing prevalence and incidence of thyroid disease in Tayside, Scotland: The Thyroid Epidemiology Audit and Research Study (TEARS). *Clinical Endocrinology*.

[2] Negro R, Mestman JH (2011). Thyroid disease in pregnancy. *Best Practice & Research*.

[3] Kasagi K, Takahashi N, Inoue G, Honda T, Kawachi Y, Izumi Y (2009). Thyroid function in japanese adults as assessed by a general health checkup system in relation with thyroid-related antibodies and other clinical parameters. *Thyroid*.

[4] Haddow JE, Palomaki GE, Allan WC (1999). Maternal thyroid deficiency during pregnancy and subsequent neuropsychological development of the child. *The New England Journal of Medicine*.

[5] Pop VJ, Kuijpens JL, Van Baar AL (1999). Low maternal free thyroxine concentrations during early pregnancy are associated with impaired psychomotor development in infancy. *Clinical Endocrinology*.

[6] Fitzpatrick DL, Russell MA (2010). Diagnosis and management of thyroid disease in pregnancy. *Obstetrics and Gynecology Clinics of North America*.

[7] Davis LE, Lucas MJ, Hankins GDV, Roark ML, Cunningham FG (1989). Thyrotoxicosis complicating pregnancy. *American Journal of Obstetrics and Gynecology*.

[8] Sheffield JS, Cunningham FG (2004). Thyrotoxicosis and heart failure that complicate pregnancy. *American Journal of Obstetrics and Gynecology*.

[9] Leung AS, Millar LK, Koonings PP, Montoro M, Mestman JH (1993). Perinatal outcome in hypothyroid pregnancies. *Obstetrics & Gynecology*.

[10] Davis LE, Leveno KJ, Cunningham FG (1988). Hypothyroidism complicating pregnancy. *Obstetrics & Gynecology*.

[11] American College of Obstetricians and Gynecologists (2002). ACOG Practice Bulletin No. 37: thyroid disease in pregnancy. *Obstetrics & Gynecology*.

[12] Tokunaga S, Sameshima H, Ikenoue T (2011). Applying the ecology model to perinatal medicine: from a regional population-based study. *Journal of Pregnancy*.

[13] Ogawa Y, Iwamura T, Kuriya N (1998). Birth size standards by gestational age for Japanese neonates. *Acta Neonatologica Japonica*.

[14] Sugaya A, Sugiyama T, Nagata M, Toyoda N (2000). Comparison of the validity of the criteria for gestational diabetes mellitus by WHO and by the Japan Society of Obstetrics and Gynecology by the outcomes of pregnancy. *Diabetes Research and Clinical Practice*.

[15] Furukawa S, Sameshima H, Ikenoue T, Ohashi M, Nagai Y (2011). Is the perinatal outcome of placental abruption modified by clinical presentation?. *Journal of Pregnancy*.

[16] Stagnaro-Green A, Abalovich M, Alexander E (2011). Guidelines of the American Thyroid Association for the diagnosis and management of thyroid disease during pregnancy and postpartum. *Thyroid*.

[17] Vaidya B, Anthony S, Bilous M (2007). Detection of thyroid dysfunction in early pregnancy: universal screening or targeted high-risk case finding?. *Journal of Clinical Endocrinology and Metabolism*.

[18] Nakova VV, Krstevska B, Dimitrovski C, Simeonova S, Hadzi-Lega M, Serafimoski V (2010). Prevalence of thyroid dysfunction and autoimmunity in pregnant women with gestational diabetes and diabetes type 1. *Prilozi*.

[19] Cleary-Goldman J, Malone FD, Lambert-Messerlian G (2008). Maternal thyroid hypofunction and pregnancy outcome. *Obstetrics & Gynecology*.

[20] Casey BM, Dashe JS, Wells CE, McIntire DD, Leveno KJ, Cunningham FG (2006). Subclinical hyperthyroidism and pregnancy outcomes. *Obstetrics & Gynecology*.

[21] Wilson KL, Casey BM, McIntire DD, Halvorson LM, Cunningham FG (2012). Subclinical thyroid disease and the incidence of hypertension in pregnancy. *Obstetrics & Gynecology*.

[22] Ashoor G, Maiz N, Rotas M, Jawdat F, Nicolaides KH (2010). Maternal thyroid function at 11 to 13 weeks of gestation and subsequent fetal death. *Thyroid*.

[23] Casey BM, Dashe JS, Wells CE (2005). Subclinical hypothyroidism and pregnancy outcomes. *Obstetrics & Gynecology*.

[24] Tudela CM, Casey BM, McIntire DD, Cunningham FG (2012). Relationship of subclinical thyroid disease to the incidence of gestational diabetes. *Obstetrics & Gynecology*.

[25] De Groot L, Abalovich M, Alexander EK (2012). Management of thyroid dysfunction during pregnancy and postpartum: an Endocrine Society clinical practice guideline. *The Journal of Clinical Endocrinology & Metabolism*.

